# Prolonged impact of the COVID-19 pandemic on self-harm hospitalizations in France: A nationwide retrospective observational study

**DOI:** 10.1192/j.eurpsy.2022.26

**Published:** 2022-06-13

**Authors:** F. Jollant, A. Roussot, E. Corruble, J. C. Chauvet-Gelinier, B. Falissard, Y. Mikaeloff, C. Quantin

**Affiliations:** 1 Department of Psychiatry and Psychotherapy, Jena University Hospital, Jena, Germany; 2 Department of psychiatry, CHU Nîmes, Nîmes, France; 3 Faculty of Medicine, Department of psychiatry, Université Paris Cité, Paris, France; 4 GHU Paris Psychiatrie et Neurosciences, Hôpital Sainte-Anne, CMME, Paris, France; 5 McGill Group for Suicide Studies, Department of psychiatry, McGill University, Montréal, Quebec, Canada; 6 Moods Research Team, INSERM UMR-1178, CESP, Le Kremlin-Bicêtre, France; 7 Department of Biostatistics and Bioinformatics (DIM), CHU Dijon, France; 8 Department of biostatistics, Université de Bourgogne Franche-Comté, Dijon, France; 9 Department of Psychiatry, CHU Bicêtre, GHU Paris-Saclay, APHP, Université Paris-Saclay, Paris, France; 10 Service de Psychiatrie et d’Addictologie, Centre Hospitalier Universitaire, Dijon, France; 11 Laboratoire de Psychopathologie et Psychologie Médicale, EA 4452, IFR Santé STIC 100, Université de Bourgogne-Franche-Comté, Dijon, France; 12 Université Paris-Saclay, UVSQ, Inserm, Developmental Psychiatry, CESP, Villejuif, France; 13 Pediatrics Department, GHU Paris-Saclay, Université Paris-Saclay, UVSQ, Inserm, Developmental Psychiatry, CESP, Villejuif, France; 14 Inserm, CIC 1432, Dijon, France; 15 Clinical Epidemiology/Clinical Trials Unit, Dijon University Hospital, Clinical Investigation Center, Dijon, France; 16 High-Dimensional Biostatistics for Drug Safety and Genomics, CESP, Université Paris-Saclay, UVSQ, Univ. Paris-Sud, Inserm, Villejuif, France

**Keywords:** Adolescents, elderly, pandemic, self-harm

## Abstract

**Background:**

The first wave of the COVID-19 pandemic in France was associated with a reduced number of hospitalizations for self-harm, with the exception of older people. The on-going pandemic may have both sustained and delayed effects.

**Methods:**

Data were extracted from the French national hospital database (PMSI), a nationwide exhaustive database. The number of self-harm hospitalizations (ICD-10 codes X60–84) between September 1, 2020 and August 31, 2021 (*N* = 85,679) was compared to 2019 (*N* = 88,782) using Poisson regression models.

**Results:**

There was a decrease in the total number of self-harm hospitalizations during the studied period versus 2019 (−3.5%; Relative Risk [RR] [95% Confidence Intervals] = 0.97 [0.96–0.97]; *p* < 0.0001). However, sex and age effects were identified. While adults aged 30–59-years-old showed a decrease (monthly decreases: −12.6 to −15.0%), we found an increase in adolescent girls (+27.7%, RR = 1.28 [1.25–1.31]; *p* < 0.0001), notably since January 2021. Moreover, the numbers were similar to 2019 in adolescent boys, in youths aged 20–29 years, and in people aged 70 and more. Hospitalizations in intensive care units decreased (−6.7%, RR = 0.93 [0.91–0.96]; *p* < 0.0001) and deaths at hospital following self-harm remained stable (+0.6%, Hazard Ratio = 0.99 [0.91–1.08], *p* = 0.79).

**Conclusions:**

During this second stage, the number of self-harm hospitalizations remained at a lower level than in the prepandemic period. However, significant variations over time, age, and sex were observed. Young people (notably adolescent girls) appear to have particularly suffered from the persistence of the pandemic, while older people did not show any decrease since the beginning. Vigilance and continuing prevention are warranted.

## Introduction

The Coronavirus 2019 disease (COVID-19) was first discovered in China at the end of 2019 and rapidly spread across the world in 2020. At the end of 2021, the COVID-19 pandemic had killed more than 5 million people worldwide and more than 290 million positive cases had been identified.

Early legitimate concerns that the COVID-19 pandemic may lead to an increased number of self-harming behaviors [1] were not realized. Indeed, initial reports showed reduced or stable rates of both suicide deaths and non-lethal self-harms during the early months of the pandemic [2, 3]. In France, an 8.5% reduction in the total number of hospitalizations for self-harm was found between March and August 2020, a decrease starting abruptly the first week of the first lockdown and persisting until the end of August when positive cases and deaths due to COVID-19 were low [4]. The decrease was observed in both men and women and in all age groups except older people.

The emergence of SARS-CoV-2 variants then caused a second wave from September to November 2020, a third wave in February–May 2021 (alpha variant), and a fourth wave in July–August 2021 (delta variant). Social distancing measures, travel restrictions, and lockdowns/curfews were reimplemented. Despite the promising start of the vaccination campaign in December 2020, national surveys showed high levels of depression, anxiety, insomnia, and suicidal ideas in the general population in France [5] and some studies additionally suggested that postlockdown periods may be marked by more severe clinical conditions [6], raising the possibility of a delayed impact on self-harm rates. Clinicians particularly alerted in media of the situation in young people and an early study reported an increasing number of visits at an emergency room in Paris in people aged 15-year-old and below starting in September 2020 [7].

The present study primarily aimed to investigate the prolonged impact of the COVID-19 pandemic on hospitalized self-harm patients by comparing the period between September 2020 to August 2021 (covering the second, third, and fourth infectious waves) with 2019 as the reference pre-COVID year. To this aim, we used a large and exhaustive national hospital database. A secondary objective was to identify similarities and differences in trends in self-harm hospitalizations between this period and the first months of the pandemic (March–August 2020) [4]. This latter objective will be presented in section “Discussion.” We hypothesized that findings from this second period would differ from the first months, notably in relation to an increasing number of self-harms in adolescents, suggesting two different stages of the pandemic in terms of effects on self-harm.

## Methods

The methods used here have been previously described in detail [4]. We used the same design as in the previous study covering the March–August 2020 period.

### Design

This is a retrospective observational study.

This manuscript follows the RECORD reporting guidelines for observational studies using routinely collected health data.

### Population

All patients aged 10 years or older, who were hospitalized for self-harm in medicine/surgery/obstetrics in France (including overseas territories) between January 1, 2019 and August 31, 2021 were included. However, the current analyses only focused on the comparison between the January–December 2019 period as the last pre-COVID comparison period, and the September 2020–August 2021 period as the studied period.

### Database

All data were extracted from the national *Programme de Médicalisation des Systèmes d’Information* (PMSI) database, an exhaustive collection of discharge summaries for all patients hospitalized in public and private hospitals in France. The authors had full access to the data.

### Variables

The main outcome was the total number of hospitalizations for self-harm in France during the period September 1, 2020 to August 31, 2021 as compared to the period January 1, 2019 to December 31, 2019 (pre-COVID period). We chose this period as the reference period (instead of September 2018–August 2019) for two reasons: (a) there are historical downwards trends in self-harm hospitalization in France so the reference period should be as close as possible to the studied period; and (b) 2019 was the reference period for our previous investigation of the March–August 2020 period [4], allowing a certain level of comparison in findings between the two COVID periods.

The outcome was analyzed by month and week, and according to 10-year age bracket and sex.

Self-harm was identified by an ICD-10 (International Classification of Diseases, 10th revision) code X60 to X84 registered as an associated diagnosis.

The secondary outcomes were the changes in the self-harming means, total number of hospital stays per patient, mean duration of hospital stays (in days) per patient, number of hospital stays in an intensive care unit, and hospital mortality following hospitalization for self-harm.

### Statistical analyses

Differences in the number of hospitalizations (per month, year, age group, sex, self-harming act) between the pre- and post-COVID periods were estimated by Relative Risks (RR) and presented with 95% Confidence Intervals [CI] using a modified Poisson regression model, as described by Zou [8] who suggested using a modified Poisson approach to estimate the relative risk and confidence intervals by using robust error variances. In our study, the robust error variances can be estimated using the SAS GENMOD procedure with the “repeated” statement. In our analyses, the subject identifier was the unique ID number of each hospital stay considered. A two-sample Student’s *t*-test was used to compare the mean number of stays and the mean length of stay per patient during the two periods. A Cox proportional-hazards regression model was applied to evaluate hospital mortality during the two periods, taking into account the length of stay for patients who died at the hospital.

The *p*-value was set a priori at 0.05 for all analyses.

All analyses were performed using SAS (SAS Institute Inc., Version 9.4, Cary, NC).

## Results

**Figure 1.**
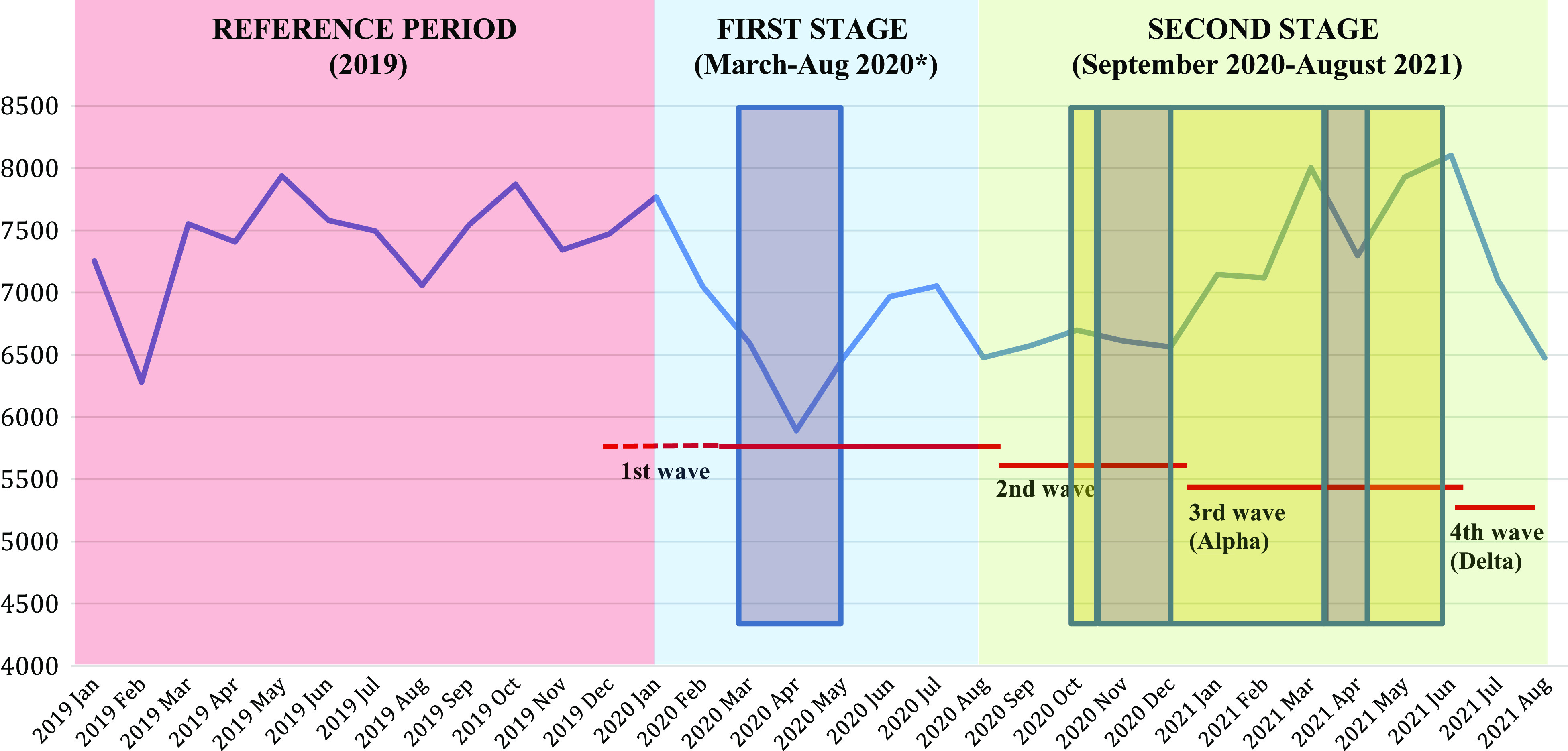
Number of monthly hospitalizations for self-harm in France in 2019, 2020, and 2021 (until August only) and representation of national confinements, curfews, and infectious waves. Red lines: infectious waves (February–August 2020 possibly starting end of 2019; September–December 2020; January–June 2021; July–August 2021). Brown squares: national confinements (March 17–May 11, 2020; October 30–December 15, 2020; March 31–May 3, 2021). Yellow squares: national curfews (October 14–October 30, 2020; December 15, 2020–March 31, 2021; May 3–June 20, 2021). Pink: Reference period (2019), Blue: first stage of the pandemic (*data available in Jollant et al. [32]); Green: Studied period (September 2020–August 2021).

**Figure 2.**
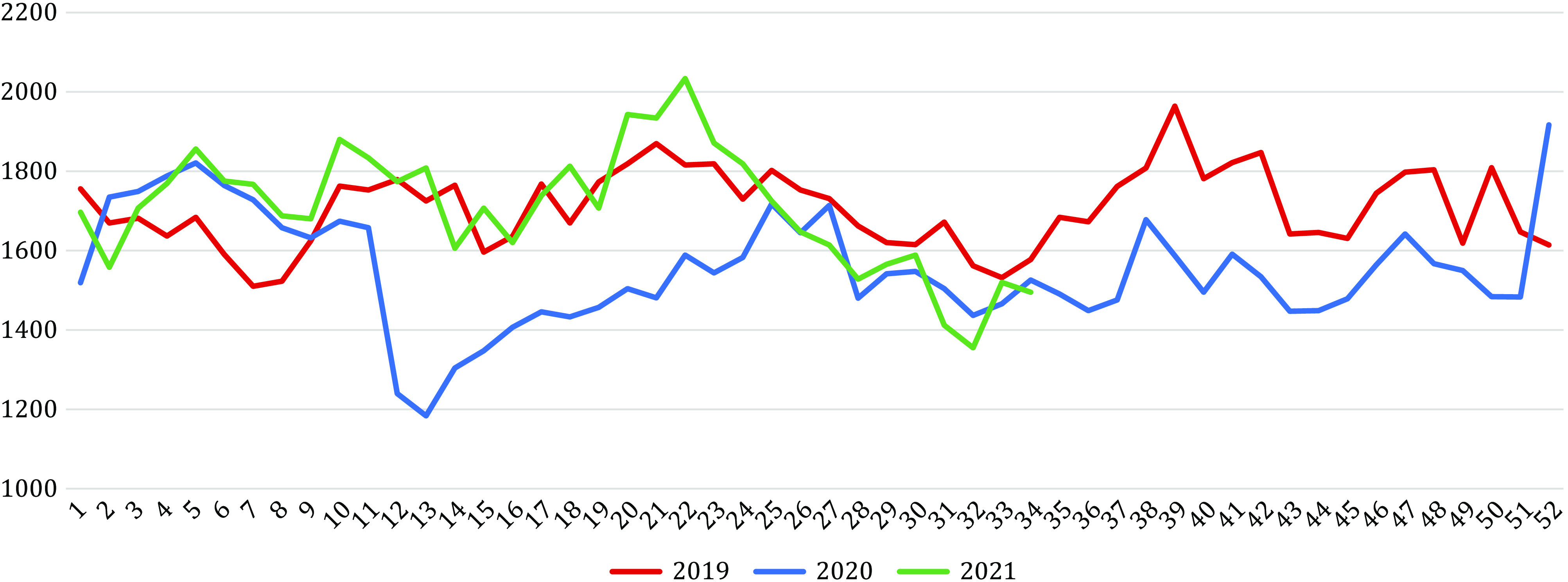
Number of weekly hospitalizations for self-harm in France in 2019, 2020, and 2021 (until August only). The number of hospitalizations at week 53 in 2020 was added to week 52 as there was official no week 53 in 2019 leading to an artificially increased number for this week.

There were a total of 228,727 hospitalizations for self-harm between January 1, 2019 and August 31, 2021, representing 180,471 patients. Weekly and monthly numbers are shown in Figures 1 and 2. As expected, this population comprised more women than men (61.3% vs. 38.7%) and the main self-harming means used were drug overdoses (72.4%) and use of sharp objects (10.7%).

### Comparison of the September 2020–August 2021 period and 2019

Overall, there were fewer self-harm hospitalizations during the September 2020–August 2021 period as compared to January–December 2019: 85,679 versus 88,782 (−3,103 representing −3.5%; RR [95% CI] = 0.97 [0.96–0.97]; *p* < 0.0001). However, the monthly changes differed over the studied period (Table 1), with a persisting decrease ranging from −10.0 to −14.9% between September and December 2020, followed by a more fluctuating pattern between January and June 2021, then a decrease in July and August 2021.

**Table 1.** Number of monthly hospitalizations for self-harm in France in September 2020–August 2021 compared to January–December 2019.

Studied period (September 2020–August 2020)	*N*	Comparison period (2019)	*N*	Difference studied versus comparison period		
				Diff. *N*	% change	Relative risk [95% CI]
September 2020	6,572	September 2019	7,542	−970	−12.9	0.87 [0.84–0.90]***
October 2020	6,699	October 2019	7,871	−1172	−14.9	0.85 [0.82–0.88]***
November 2020	6,610	November 2019	7,343	−733	−10.0	0.90 [0.87–0.93]***
December 2020	6,562	December 2019	7,470	−908	−12.2	0.88 [0.85–0.91]***
January 2021	7,147	January 2019	7,251	−104	−1.4	0.99 [0.95–1.02]
February 2021	7,129	February 2019	6,280	849	13.5	1.14 [1.10–1.17]***
March 2021	8,006	March 2019	7,551	455	6.0	1.06 [1.03–1.09]**
April 2021	7,300	April 2019	7,406	−106	−1.4	0.99 [0.95–1.02]
May 2021	7,927	May 2019	7,936	−9	−0.1	1.00 [0.97–1.03]
June 2021	8,108	June 2019	7,581	527	7.0	1.07 [1.04–1.10]***
July 2021	7,122	July 2019	7,495	−373	−5.0	0.95 [0.92–0.98]*
August 2021	6,497	August 2019	7,056	−559	−7.9	0.92 [0.89–0.95]***

*Note*: Relative risk from Poisson regression model. For January–August 2020 versus 2019 numbers and statistics, please see Jollant et al. [32]. Lancet Regional Health Europe 2021.

Abbreviation: CI, confidence interval.

* *p* < 0.05;

** *p* ≤ 0.001;

*** *p* ≤ 0.0001.

Differences were observed by sex, with a significant decrease found during the studied period versus 2019 for men (−8.0%; RR = 0.92 [0.91–0.93]; *p* < 0.0001) but not women (−0.6%; RR = 0.99 [0.98–1.01]; *p* = 0.3) (Table 2). Men represented 88.9% of the global decrease in numbers during this period.

**Table 2.** Number of hospitalizations for self-harm in France in September 2020–August 2021 compared to January–December 2019, per age group and sex.

Age group	*N* in 2019 (comparison period)			*N* in September 2020–August 2021 (studied period)			Difference in *N* studied versus comparison period			% change			Relative risk [95% CI]		
	Men	Women	Total	Men	Women	Total	Men	Women	Total	Men	Women	Total	Men	Women	Total
10–19	3,808	13,445	17,253	3,794	17,170	20,964	−14	3,725	3,711	−0.4	27.7	21.5	1.00 [0.95–1.04]	1.28 [1.25–1.31]***	1.22 [1.19–1.24]***
20–29	5,748	7,921	13,669	5,591	8,033	13,624	−157	112	−45	−2.7	1.4	−0.3	0.97 [0.94–1.01]	1.01 [0.98–1.05]	1.00 [0.97–1.02]
30–39	6,768	6,854	13,622	5,934	5,716	11,650	−834	−1,138	−1,972	−12.3	−16.6	−14.5	0.88 [0.85–0.91]***	0.83 [0.81–0.86]***	0.86 [0.83–0.88]***
40–49	7,315	9,000	16,315	6,506	7,369	13,875	−809	−1,631	−2,440	−11.1	−18.1	−15.0	0.89 [0.86–0.92]***	0.82 [0.79–0.84]***	0.85 [0.83–0.87]***
50–59	5,899	8,301	14,200	5,132	7,276	12,408	−767	−1,025	−1,792	−13.0	−12.3	−12.6	0.87 [0.84–0.90]***	0.88 [0.85–0.91]***	0.87 [0.85–0.90]***
60–69	2,676	4,401	7,077	2,606	4,045	6,651	−70	−356	−426	−2.6	−8.1	−6.0	0.97 [0.92–1.03]	0.92 [0.88–0.96]**	0.94 [0.91–0.97]**
70–79	1,301	2,274	3,575	1,278	2,208	3,486	−23	−66	−89	−1.8	−2.9	−2.5	0.98 [0.91–1.06]	0.97 [0.92–1.03]	0.98 [0.93–1.02]
80+	1,200	1,871	3,071	1,114	1,907	3,021	−86	36	−50	−7.2	1.9	−1.6	0.93 [0.86–1.01]	1.02 [0.96–1.09]	0.98 [0.94–1.03]
Total	34,715	54,067	88,782	31,955	53,724	85,679	−2,760	−343	−3,103	−8.0	−0.6	−3.5	0.92 [0.91–0.93]***	0.99 [0.98–1.01]	0.97 [0.96–0.97]***

*Note:* Relative risk from Poisson regression model.

Abbreviation: CI, confidence interval.

* *p* < 0.05;

** *p* ≤ 0.001;

*** *p* ≤ 0.0001.

**Figure 3.**
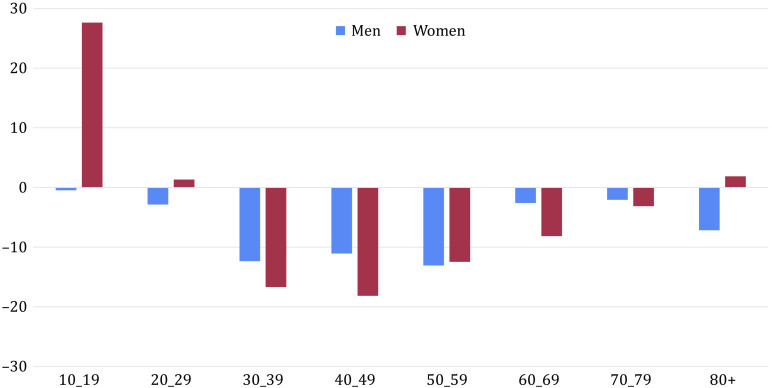
Changes (in %) in the number of hospitalizations for self-harm in France in September 2020–August 2021 compared to January–December 2019, per age group and by sex.

A significant effect of age was also found (Table 2 and Figure 3). There was a large increase in self-harm hospitalizations in women aged 10–19 years (+27.7%, RR = 1.28 [1.25–1.31]; *p* < 0.0001); and a stability in numbers in male adolescents aged 10–19, in both sexes among 20–29-year-olds, and in people above 70. All other age groups showed decreasing trends during the studied period versus 2019. Large decreases were notably found for middle-aged adults (30–59-years-old) in both men and women.

**Figure 4.**
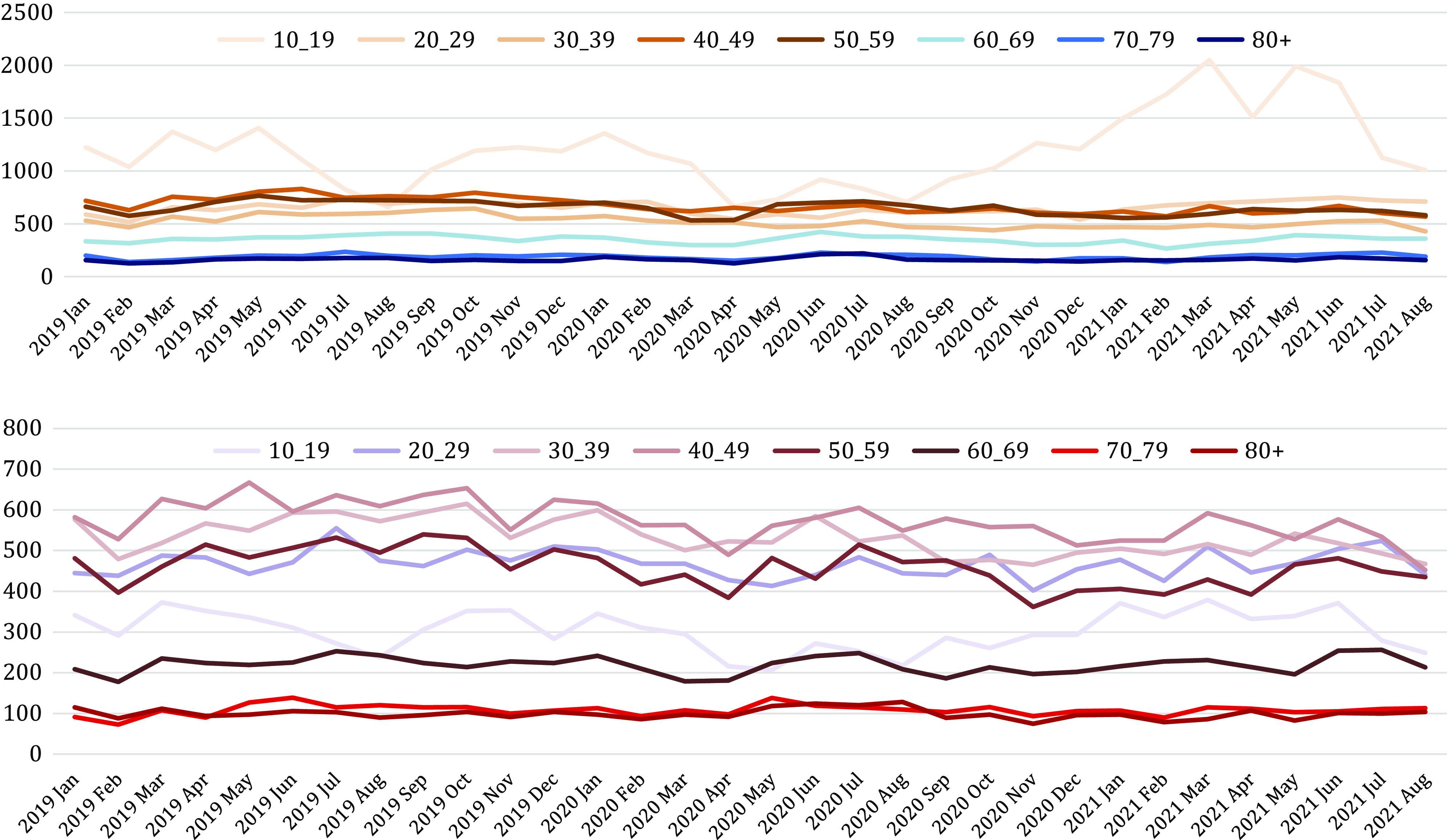
Number of monthly hospitalizations for self-harm in France in 2019, 2020, and 2021 (until August only) per age group in women (top) and men (bottom).

When focusing on 10–19-year-old female adolescents, the annual trends in self-harm hospitalizations (Figure 4) showed the expected seasonal pattern (lowest in summer, highest in winter and spring), but a more marked increase during winter and spring 2021 versus 2019. It was followed by a decrease during summer 2021, although the number of hospitalizations during the 2021 summer period remained higher than in summer 2019 and summer 2020.

All types of self-harm means significantly decreased or were stable as compared to 2019, except for use of sharp objects and jumping from height (Table 3). In female adolescents aged 10–19, significant increases were found for use of sharp objects (+48.9%; RR = 1.49 [1.43–1.56]; *p* < 0.0001), drug overdose (+18.4%; 1.18 [1.15–1.22]; *p* < 0.0001), alcohol use (+26.5%; 1.27 [1.04–1.53]; *p* < 0.05), use of chemicals (+19.2%; 1.19 [1.01–1.40]; *p* < 0.05), hanging and strangulation (+48.7%; 1.49 [1.26–1.76]; *p* < 0.0001), jumping from height (+28.8%; 1.29 [1.05–1.57], *p* < 0.05), and use of other/unspecified means (+45.2%; 1.45 [1.30–1.63]; *p* < 0.0001).

**Table 3.** Number of hospitalizations for self-harm in France in September 2020–August 2021 compared to January–December 2019, according to the self-harm means and characteristics of hospital stays.

	*N* in 2019	*N* in September 2020–August 2021	Difference in *N*	% change	Relative risk [95% CI] or *t*-test
*Self-harm means*					
Drugs (X60–X64)	65,078	60,850	−4,228	−6.5	0.94 [0.92–0.95]** *
Alcohol (X65)	4,170	3,946	−224	−5.4	0.95 [0.91–0.99]*
Additional means (X66–X82), including:	16,085	17,927	1,842	11.5	1.11 [1.09–1.14]** *
Solvents, carbon monoxyde, pesticides, other chemicals (X66–X69)	2,228	2,274	46	2.1	1.02 [0.96–1.08]
Hanging, strangulation (X70)	2,556	2,579	23	0.9	1.01 [0.96–1.07]
Drowning (X71)	206	224	18	8.7	1.09 [0.90–1.31]
Firearm (X72–X74)	462	452	−10	−2.2	0.98 [0.86–1.11]
Fire, explosive (X75–X77)	298	269	−29	−9.7	0.90 [0.77–1.06]
Sharp or blunt object (X78–X79)	8,677	10,403	726	19.9	1.2 [1.17–1.23]** *
Jumping from height (X80)	1,410	1,516	106	7.5	1.08 [1.00–1.16]*
Jumping/crashing (X81–X82)	248	210	−38	−15.3	0.85 [0.70–1.02]
Other and undefined means (X83–X84)	3,449	2,956	−493	−14.3	0.86 [0.82–0.90]** *
*Characteristics of hospital stays*					
Patients (*N*)	75,988	72,216	−3,772	−5.0	0.95 [0.94–0.96]** *
Hospital stays per patient (*N* [SD])	1.17 (±0.66)	1.19 (±0.86)	—	—	*t*-test = −4.54** £
Average length of stay (days [SD])	3.07 (± 7.93)	3.24 (± 7.63)	—	—	*t*-test = −4.74** £
Hospitalization in intensive care	8,630	8,055	−575	−6.7	0.93 [0.91–0.96]** *
*Death at discharge*	1,025	1,031	6	0.6	0.99 [0.91–1.08]§

*Note:* Relative risk from Poisson regression model; £, Student *t*-test; §, Cox model.

Abbreviations: CI, confidence interval; SD, standard deviation.

* *p* < 0.05;

** *p* ≤ 0.001;

*** *p* ≤ 0.0001.

The number of hospitals stays per patient and the hospitalization duration slightly increased during the studied period versus 2019.

Finally, a significant global decrease in self-harm hospitalization in intensive care units was found compared to 2019, while no change in the number of deaths in hospital following self-harm was observed (Table 3). In adolescent girls, however, the number of intensive care unit hospitalizations increased during the studied period versus 2019 (+20.6%; RR = 1.21 [1.05–1.39]; *p* < 0.05).

## Discussion

### General findings and comparison with the first months of the pandemic

As compared to 2019 (the last pre-COVID year), the COVID-19 pandemic during the period September 2020–August 2021—covering the second, third, and fourth infectious waves—showed different patterns in self-harm hospitalizations compared to the early months of the pandemic and the first infectious wave (March–August 2020) [4] (see summary Table 4). First, the global decreasing trends observed during the first stage of the pandemic persisted but at a lower intensity. This was particularly the case for 30–59-year-old adults until the end of the studied period in August 2021. Second, young people (10–29-years-old) showed a very different temporal pattern, suggesting a delayed effect. The decrease in self-harm hospitalizations observed in these age groups during the first pandemic stage was replaced by (a) a large increase in hospitalized self-harms in teenage girls (notably between January and June 2021) and the use of more violent acts in this population, and (b) similar levels of self-harming behaviors as compared to 2019 in 10–19-year-old boys and in 20–29-year-olds in both sexes. Third, people above 70-years-old continued to show similar levels of hospitalized self-harm as in 2019, with no decrease since the start of the pandemic. Finally, hospitalization in intensive care units decreased (with the notable exception of teenage girls) and deaths at hospital were comparable to 2019, suggesting no global negative impact of the pandemic on more lethal acts to date.

**Table 4.** Summary of findings by age group and gender during the first (March–August 2020, Jollant et al. [32]) and second (September 2020–August 2021) stages versus 2019.

Group	March–August 2020 versus 2019	September 2020–August 2021 versus 2019
Adolescents		
Females	Decreased	Increased
Males	Decreased	Stable
Young adults		
Females	Decreased	Stable
Males	Decreased	Stable
Middle-aged people		
Females	Decreased	Decreased
Males	Decreased	Decreased
Old-aged people		
Females	Stable or increased	Stable
Males	Stable or increased	Stable

### A global decrease in self-harm hospitalizations

Concerns that the prolongation of the pandemic may be associated with significant increases in the number of self-harming behaviors were not realized. The overall decrease in hospitalized self-harms observed during the first stage (−8.5%) [4] continued, although at a lower level (−3.5%). This was notably the case for middle-aged individuals in both men and women. Among 30–59-year-olds, the decreases in the number of self-harm hospitalizations during the studied period as compared to 2019 ranged from −12.6 to −15.0%. A decrease of −6% was also found in people aged 60–69 years old. Similar findings have been found in Northern England regarding primary care recorded self-harm, with a stronger decrease until August 2020 followed by a weaker decrease until May 2021 [9]. However, other locations have observed increasing trends during the first months of the pandemic [6, 10].

It is remarkable that these decreases persisted at such high rates. Indeed, results from phone surveys conducted by Public Health France [5] in a representative sample of the French adult general population (therefore, mainly middle-aged people) reported high rates of depression (18%), anxiety (23%), sleep problems (68%), and suicidal ideas (10%) at levels superior to the pre-COVID period. Similar results were found in a systematic review of studies conducted in 204 countries, demonstrating a significant increase in depressive and anxiety disorders, especially among women, since the start of the pandemic [11]. A study in Sydney, Australia, also reported a discordance between increased numbers of presentation at hospital for suicidal ideation but decreased number of self-harms during the first 15 months of the pandemic [12]. These findings highlight, if needed, the importance of monitoring depressive/anxiety symptoms, suicidal ideation, self-harming acts, and suicide deaths separately.

A speculative explanation may be that economic measures taken by the French government, including financial support for endangered professional activities such as restaurants or touristic places, and state-guaranteed loans for companies, may have limited the negative impact of the pandemic on working populations. Moreover, the national economy showed an unexpected rapid recovery (as early as the third trimester of 2020) in terms of unemployment and gross domestic product. Previous studies have shown that active labor market programs limit the impact on suicides during economic crises [13].

### The case of adolescents and young adults

In young people, two phenomena were observed. Adolescent girls showed a large increase in self-harm hospitalizations (+27.7%), while adolescent boys and 20–29-year-old men and women showed stable numbers as compared to 2019. This is in sharp contrast to 30–59-year-old adults during the same period, and to the same age groups during the first stage of the COVID-19 pandemic (−5.0 to −22.1% in France [4]). The increase observed in teenage girls in France since January 2021 seems to follow the usual pre-COVID seasonal pattern that is, increasing trends during winter and spring followed by decreasing trends during the summer vacation (July–August). However, it was much more marked in 2021 than in 2019. Additional worrying observations were the increase in the use of more violent self-harming means such as hanging, jumping from height, or use of chemicals, and the increase in intensive care unit hospitalizations in female adolescents.

This temporal evolution in self-harming behaviors in adolescent females during the COVID-19 pandemic was also reported in the USA: after an initial decrease, a 26.2% increase in the number of visits to the emergency departments for suicide attempt was observed in girls aged 12–17 years. This phenomenon started in early May 2020 and was still observed in May 2021 [14]. In Manchester, England, an increase in self-harms in adolescents aged 10–17 was also observed since August 2020 [9]. In Ontario, Canada, a similar initial decline in self-harms in young people was also found [15] followed by an increase in adolescents during the second infectious wave in September 2020–January 2021 [16].

Explanations are only speculative so far. First, the importance of social interactions for biopsychosocial development [17, 18] might render young people particularly sensitive to impairments in social life, including in the domains of family, school/university, romantic relationships, and friendships. A study in Switzerland during the first lockdown showed that the main sources of perceived stress among adolescents were the disruption of social life and main activities, notably among young women [19]. The same study also underlined high rates of excessive internet use. In France, the studied period comprised several weeks of lockdowns or curfews, and the limitation in the sizes of recreational, festive and familial gatherings that were only raised in summer 2021. Moreover, there were numerous temporary classroom closures, and closure of universities for several weeks during the 2020–2021 academic year. Finally, lockdown periods may have had negative impacts on the functioning of many families [20], including high rates of domestic violence toward juveniles [16, 21, 22].

This situation may have increased the risk of mental disorders among young people. Several studies have highlighted the increasing prevalence of depression in young generations before the start of the pandemic [23]. The pandemic may have exacerbated this phenomenon. For instance, a study among workers showed higher changes in severity and prevalence of depressive and anxious symptoms during the COVID-19 pandemic among the youngest versus oldest workers [24]. A literature review revealed increasing rates of depression and anxiety among adolescents since COVID-19, notably in girls [25]. A Canadian survey among 12–18-year-old adolescents found a significant association between deliberate self-harm during the pandemic and (a) self-identification as transgender, nonbinary, or gender fluid; (b) not residing with both parents; and (c) psychiatric concerns or frequent cannabis use [26]. It is therefore possible that the pandemic context and related effects on social life had a particularly strong impact on the mental health of younger generations.

One last speculative explanation may be that the young generations are currently exposed to high levels of uncertainty and hopelessness regarding their future, related notably to geopolitical tensions in the world and major climate changes, with limited demonstrations that things will improve any soon.

### The case of older people

People aged 70-years-old and more showed no change in the number of self-harm hospitalizations during the studied period in comparison to 2019 contrary to middle-age adults. Moreover, this observation was also previously found in the first stage of the pandemic [4]. Literature on self-harm in older people during the pandemic remains scarce. A study of suicide -related calls to a national crisis hotline in Israel during the early months of the pandemic found a 30-fold increase in people aged 50 and more [27].

Older people may have been particularly affected by the COVID-19 pandemic at three levels. First, they were the age group at the highest risk of COVID-19 mortality since the beginning of the pandemic and during the whole studied period. Second, they may have particularly suffered from social contact restrictions due to limited use of virtual ways of communications, the absence of professional interactions, and sometimes living alone. A study in Hong-Kong during the COVID pandemic found an association between loneliness and suicidal ideation in older people with late-life depression [28]. Finally, the COVID pandemic led to an important reduction in access to care for both physical and mental health, as shown by an English population-based study [29]. This may have particularly affected this population.

Until statistics are published, it cannot be fully excluded that the numbers of suicide deaths increased during the pandemic in this age group characterized by a low suicide attempt on suicide deaths ratio [30]. For instance, a study in Taiwan showed age differences in suicide rates during the early months of the pandemics, with an initial decrease followed by an increase in older people [31]. More research in this specific age group is urgent and vigilance is needed.

### About more lethal acts

Finally, our study did not identify any impact of COVID-19 pandemic on more lethal acts (with the exception of more intensive care unit hospitalizations in teenage girls). Contrary to initial concerns during the first stage [4], intensive care hospitalizations decreased, and deaths in hospitals following self-harm were stable compared with 2019. This is in line with the international literature mainly showing stable or decreasing numbers of suicide deaths during the early months of the pandemic [3]. National data on suicide mortality in France are unfortunately not yet available for the pandemic period. More recent observations remain limited and continued vigilance is warranted.

### Limitations

Several limitations should be kept in mind. First, this study is limited to self-harming behaviors leading to a hospitalization. It therefore does not include those leading to an emergency department visit without subsequent hospitalization or acts with no hospital presentation [32]. However, findings are largely in line with those from calls to poison control centers for a suicide attempt [32], and with those from emergency department visits (unpublished data from *Santé Publique France*) suggesting that our findings are not due to, for instance, limited bed availability. Second, it cannot be excluded that many hospitalizations following self-harm were not accurately coded as self-harm. However, this would have affected each year unless there were changes in coding procedures between 2019 and 2021, which cannot be fully confirmed or excluded. The fact that similar findings have been reported in different countries tend to support a global phenomenon. Third, hospital databases do not have extensive information, limiting the interpretation of findings. For instance, conditions of living, or professional or financial information are not found here. Additional clinical studies are required to understand the mechanisms behind the phenomena reported here, notably in terms of age and sex effects.

## Data Availability

The PMSI database was transmitted by the national agency for the management of hospitalization data (ATIH number 2015-111111-47-33), which manages this sensitive information (https://www.atih.sante.fr/), and cannot be directly shared.

## References

[1] Gunnell D, Appleby L, Arensman E, Hawton K, John A, Kapur N, et al. Suicide risk and prevention during the COVID-19 pandemic. Lancet Psychiatry. 2020;7(6):468–71. 32330430 10.1016/S2215-0366(20)30171-1 PMC7173821

[2] Hawton K, Casey D, Bale E, Brand F, Ness J, Waters K, et al. Self-harm during the early period of the COVID-19 pandemic in England: comparative trend analysis of hospital presentations. J Affect Disord. 2021;282:991–5. 33601744 10.1016/j.jad.2021.01.015 PMC7832687

[3] Pirkis J, John A, Shin S, DelPozo-Banos M, Arya V, Analuisa-Aguilar P, et al. Suicide trends in the early months of the COVID-19 pandemic: an interrupted time-series analysis of preliminary data from 21 countries. Lancet Psychiatry. 2021;8:P579–88. 10.1016/S2215-0366(21)00091-2 PMC9188435 33862016

[4] Jollant F, Roussot A, Corruble E, Chauvet-Gelinier JC, Falissard B, Mikaeloff Y, et al. Hospitalization for self-harm during the early months of the COVID-19 pandemic in France: a nationwide retrospective observational cohort study. Lancet Reg Health Eur. 2021;6:100102. 34557830 10.1016/j.lanepe.2021.100102 PMC8454825

[5] Santé Publique France. Étude CoviPrev; 2021.

[6] Ambrosetti J, Macheret L, Folliet A, Wullschleger A, Amerio A, Aguglia A, et al. Psychiatric emergency admissions during and after COVID-19 lockdown: short-term impact and long-term implications on mental health. BMC Psychiatry. 2021;21(1):465. 34560856 10.1186/s12888-021-03469-8 PMC8464091

[7] Cousien A, Acquaviva E, Kernéis S, Yazdanpanah Y, Delorme R. Temporal trends in suicide attempts among children in the decade before and during the COVID-19 pandemic in Paris, France. JAMA Netw Open. 2021;4(10):e2128611. 34618041 10.1001/jamanetworkopen.2021.28611 PMC8498848

[8] Zou G. A modified poisson regression approach to prospective studies with binary data. Am J Epidemiol. 2004;159(7):702–6. 15033648 10.1093/aje/kwh090

[9] Steeg S, Bojanić L, Tilston G, Williams R, Jenkins DA, Carr MJ, et al. Temporal trends in primary care-recorded self-harm during and beyond the first year of the COVID-19 pandemic: time series analysis of electronic healthcare records for 2.8 million patients in the Greater Manchester Care Record. EClinicalMedicine. 2021;41:101175. 34746726 10.1016/j.eclinm.2021.101175 PMC8557994

[10] Beghi M, Ferrari S, Biondi L, Brandolini R, Corsini C, De Paoli G, et al. Mid-term psychiatric consequences of the COVID-19 pandemic: a 4 months observational study on emergency room admissions for psychiatric evaluation after the (first) lockdown period in Italy. Soc Psychiatry Psychiatr Epidemiol. 2022;57:1283–9. 35279745 10.1007/s00127-022-02262-6 PMC8917958

[11] COVID-19 MDC. Global prevalence and burden of depressive and anxiety disorders in 204 countries and territories in 2020 due to the COVID-19 pandemic. Lancet 2021;398(10312):1700–12. 34634250 10.1016/S0140-6736(21)02143-7 PMC8500697

[12] Sperandei S, Page A, Bandara P, Reis A, Saheb R, Gaur P et al. The impact of the COVID-19 pandemic on hospital-treated self-harm in Sydney (Australia). Aust N Z J Psychiatry. 2022:48674211068393. 34996305 10.1177/00048674211068393

[13] Stuckler D, Basu S, Suhrcke M, Coutts A, McKee M. The public health effect of economic crises and alternative policy responses in Europe: an empirical analysis. Lancet. 2009;374(9686):315–23. 19589588 10.1016/S0140-6736(09)61124-7

[14] Yard E, Radhakrishnan L, Ballesteros MF, Sheppard M, Gates A, Stein Z, et al. Emergency department visits for suspected suicide attempts among persons aged 12-25 years before and during the COVID-19 pandemic - United States. MMWR Morb Mortal Wkly Rep. 2021;70(24):888–94. 34138833 10.15585/mmwr.mm7024e1 PMC8220953

[15] Ray JG, Austin PC, Aflaki K, Guttmann A, Park AL. Comparison of self-harm or overdose among adolescents and young adults before vs during the COVID-19 pandemic in Ontario. JAMA Netw Open. 2022;5(1):e2143144. 35019981 10.1001/jamanetworkopen.2021.43144 PMC8756304

[16] Stewart SL, Vasudeva AS, Van Dyke JN, Poss JW. Following the epidemic waves: child and youth mental health assessments in Ontario through multiple pandemic waves. Front Psychiatry. 2021;12:730915. 34867522 10.3389/fpsyt.2021.730915 PMC8635704

[17] Gifuni AJ, Chakravarty MM, Lepage M, Ho TC, Geoffroy MC, Lacourse E, et al. Brain cortical and subcortical morphology in adolescents with depression and a history of suicide attempt. J Psychiatry Neurosci. 2021;46(3):E347–57. 33961355 10.1503/jpn.200198 PMC8327980

[18] Casey BJ, Jones RM, Hare TA. The adolescent brain. Ann N Y Acad Sci. 2008;1124:111–26. 18400927 10.1196/annals.1440.010 PMC2475802

[19] Mohler-Kuo M, Dzemaili S, Foster S, Werlen L, Walitza S. Stress and mental health among children/adolescents, their parents, and young adults during the first COVID-19 lockdown in Switzerland. Int J Environ Res Public Health. 2021;18(9):4668. 33925743 10.3390/ijerph18094668 PMC8124779

[20] Hwang P, Ipekian L, Jaiswal N, Scott G, Amirali EL, Hechtman L. Family functioning and mental wellbeing impairment during initial quarantining for the COVID-19 pandemic: a study of Canadian families. Curr Psychol. 2022:1–13. 10.1007/s12144-021-02689-1 PMC8743689 35035192

[21] Loiseau M, Cottenet J, Bechraoui-Quantin S, Gilard-Pioc S, Mikaeloff Y, Jollant F, et al. Physical abuse of young children during the COVID-19 pandemic: alarming increase in the relative frequency of hospitalizations during the lockdown period. Child Abuse Negl. 2021;122:105299. 34488053 10.1016/j.chiabu.2021.105299 PMC8435815

[22] de Oliveira SMT, Galdeano EA, da Trindade EMGG, Fernandez RS, Buchaim RL, Buchaim DV, et al. Epidemiological study of violence against children and its increase during the COVID-19 pandemic. Int J Environ Res Public Health. 2021;18(19):10061. 34639362 10.3390/ijerph181910061 PMC8507936

[23] Hidaka BH. Depression as a disease of modernity: explanations for increasing prevalence. J Affect Disord. 2012;140(3):205–14. 22244375 10.1016/j.jad.2011.12.036 PMC3330161

[24] Thomas D, Lawton R, Brown T, Kranton R. Prevalence, severity and distribution of depression and anxiety symptoms using observational data collected before and nine months into the COVID-19 pandemic. Lancet Reg Health Am. 2021;1:100009. 34514462 10.1016/j.lana.2021.100009 PMC8421709

[25] Racine N, McArthur BA, Cooke JE, Eirich R, Zhu J, Madigan S. Global prevalence of depressive and anxiety symptoms in children and adolescents during COVID-19: a meta-analysis. JAMA Pediatr. 2021;175:1142–50. 34369987 10.1001/jamapediatrics.2021.2482 PMC8353576

[26] Turner BJ, Robillard CL, Ames ME, Craig SG. Prevalence and correlates of suicidal ideation and deliberate self-harm in Canadian adolescents during the coronavirus disease 2019 pandemic. Can J Psychiatry. 2021;67:403–6. 34378420 10.1177/07067437211036612 PMC9065494

[27] Zalsman G, Levy Y, Sommerfeld E, Segal A, Assa D, Ben-Dayan L, et al. Suicide-related calls to a national crisis chat hotline service during the COVID-19 pandemic and lockdown. J Psychiatr Res. 2021;139:193–6. 34087516 10.1016/j.jpsychires.2021.05.060 PMC8769684

[28] Louie LLC, Chan WC, Cheng CPW. Suicidal risk in older patients with depression during COVID-19 pandemic: a case-control study. East Asian Arch Psychiatr. 2021;31(1):3–8. 10.12809/eaap2055 33753570

[29] Mansfield KE, Mathur R, Tazare J, Henderson AD, Mulick AR, Carreira H, et al. Indirect acute effects of the COVID-19 pandemic on physical and mental health in the UK: a population-based study. Lancet Digit Health. 2021;3(4):e217–30. 33612430 10.1016/S2589-7500(21)00017-0 PMC7985613

[30] Beghi M, Butera E, Cerri CG, Cornaggia CM, Febbo F, Mollica A, et al. Suicidal behaviour in older age: a systematic review of risk factors associated to suicide attempts and completed suicides. Neurosci Biobehav Rev. 2021;127:193–211. 33878336 10.1016/j.neubiorev.2021.04.011

[31] Chen YY, Yang CT, Pinkney E, Yip PSF. Suicide trends varied by age-subgroups during the COVID-19 pandemic in 2020 in Taiwan. J Formos Med Assoc. 2022;121:1174–77. 34674903 10.1016/j.jfma.2021.09.021 PMC8493279

[32] Jollant F, Hawton K, Vaiva G, Chan-Chee C, du Roscoat E, Leon C. Non-presentation at hospital following a suicide attempt: a national survey. Psychol Med. 2020;52:1–8. 10.1017/S0033291720002305 32618240

